# Myasthenia gravis and independent risk factors for recurrent infection: a retrospective cohort study

**DOI:** 10.1186/s12883-023-03306-3

**Published:** 2023-07-03

**Authors:** Chia-Yin Chien, Chun-Wei Chang, Ming-Feng Liao, Chun-Che Chu, Long-Sun Ro, Yih-Ru Wu, Kuo-Hsuan Chang, Chiung-Mei Chen, Hung-Chou Kuo

**Affiliations:** 1 Department of Neurology, Chang Gung Memorial Hospital Linkou Medical Centre, Taoyuan, Taiwan; 2 College of Medicine, Chang Gung University, Taoyuan, Taiwan; 3 Department of Neurology, Chang Gung Memorial Hospital & Chang Gung University, No. 5, Fuxing St, Guishan Dist, Taoyuan City, 333423 Taiwan

**Keywords:** Myasthenia gravis, Myasthenic crisis, Reinfection, Risk factors, Diabetes mellitus

## Abstract

**Background:**

Approximately 10% to 20% of myasthenia gravis (MG) patients have experienced a myasthenic crisis (MC), which contributes to morbidity and mortality. MC triggered by infection is associated with poor outcomes. However, there is a lack of prognostic factors that clinicians can utilize to target interventions for preventing recurrent infection-triggered MC. This study aimed to characterize clinical manifestations, comorbidities, and biochemical profiles associated with recurrent infection-triggered MC in MG patients.

**Methods:**

This retrospective study included 272 MG patients hospitalized with an infection requiring at least 3 days of antibiotics from January 2001 to December 2019. Patients were further stratified into non-recurrent or recurrent infection groups. Clinical features such as gender, age, concomitant diseases, acetylcholine receptor antibodies and biochemical data (including electrolytes and coagulants), muscle strength of pelvic and shoulder girdle, bulbar and respiratory function, management with an endotracheal tube, Foley catheter, or plasmapheresis, duration of hospitalization, and culture pathogens were recorded.

**Results:**

The recurrent infection group was significantly older than the non-recurrent group (median age, 58.5 versus 52.0 years). Pneumonia was the most common infection and *Klebsiella pneumoniae* was the most common pathogen. The presence of concomitant diabetes mellitus, activated partial thromboplastin time prolongation, the duration of hospitalization, and hypomagnesaemia were independently associated with recurrent infection. The presence of deep vein thrombosis, thymic cancer, and electrolyte imbalances i.e., hypokalemia, and hypoalbuminemia were significantly associated with a risk for infection. The influence of endotracheal intubation, anemia, and plasmapheresis during hospitalization were inconsistent.

**Conclusions:**

The independent risk factors for recurrent infections in MG patients identified in this study include the presence of concomitant diabetes mellitus, hypomagnesaemia, activated partial thromboplastin time prolongation, and longer duration of hospitalization, highlighting the need for targeted interventions to prevent recurrent infections in this population. Further research and prospective studies are warranted to validate these findings and refine interventions for optimizing patient care.

**Supplementary Information:**

The online version contains supplementary material available at 10.1186/s12883-023-03306-3.

## Background

Myasthenia gravis (MG) is one of the major autoimmune neuromuscular disorders [1]. Acetylcholine receptor (AChR) antibodies, which are the major specific autoantibodies detected in around 30% to 50% of patients with ocular MG (OMG) and 85% of patients with generalized MG (GMG), lead to tissue and functional damage at the neuromuscular junction [2–5]. On the other hand, 4% to 7% of all MG cases are associated with muscle specific kinase (MuSK) antibodies [1, 5, 6].

Myasthenic crisis (MC) or class V according to the Myasthenia Gravis Foundation of America (MGFA) classification, is usually associated with GMG [1, 7]. It presents as an exacerbation of bulbar and respiratory weakness, leading to nasogastric intubation, endotracheal intubation, or mechanical ventilation [8–10]. Approximately 10% to 20% of MG patients have experienced a MC, which contributes to worsening ability to perform activities of daily living as well as increasing the risk of mortality [6, 9, 11, 12]. Several potential factors have been observed to trigger a MC manifestation including infections, adverse effects of medication, co-morbidity, or tapering of immunosuppressive agents. Infections, particularly pneumonia or upper respiratory infections, account for 40% to 70% of cases [7, 13]. In addition, MC triggered by infection is associated with poor outcomes and imposes a significant disease burden on affected patients [9, 13]. It is therefore critical to identify risk factors for infection in order to prevent MC and reduce the associated disease burden.

Some researches have reported that higher MGFA class, the absence of AChR antibodies, certain comorbidities and infections, or inappropriate therapeutic strategies can be potential predictors for an exacerbation of MC [4, 9]. Some studies otherwise focused on the prognostic factors of MC among MG patients with thymectomy or not [9, 14]. However, risk factors for recurrent infection-triggered MC remain largely unknown and there is a lack of prognostic factors that clinicians can utilize to target interventions for preventing recurrent infection-triggered MC. This study aimed to characterize factors predictive of recurrent infection-triggered MC, by exploring the clinical manifestation and laboratory results of patients with MG hospitalized with an infection.

## Materials and methods

### Patient recruitment

**Fig. 1:**
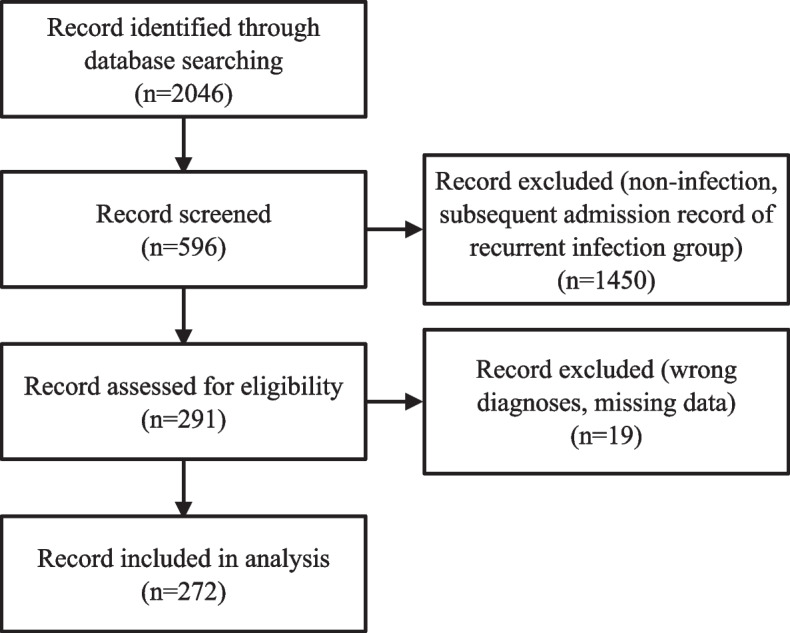
Flowchart detailing the screening and inclusion of patient records for this study. Missing data were those with no records on the verbal status of Glasgow coma scale nor on the sum score of both deltoid and iliacus muscles strength according to Medical Research Council scale of Great Britain

This study was approved by the Institutional Review Board of Chang Gung Memorial Hospital, Taiwan (202300114B0(2,301,130,050)). We retrospectively identified MG inpatients from January 2001 to December 2019 using a computerized search of the Chang Gung Research Database (Supplementary Table 1). Infection was defined as hospitalization with systemic antibiotics for more than three days [15, 16]. Respiratory tract cultures, urine cultures, and blood cultures represented the presence of pneumonia, urinary tract infection and bacteremia, separately [15, 16]. Multiple infections were counted if there was more than one positive culture in the same patient. The selected MG patients with an infection were further classified into a non-recurrent (only one infection episode) or recurrent (more than one infection episode) group. For those who had multiple hospitalization within the recruitment period, only the first record was included. All medical records were reviewed to confirm the final diagnosis of MG based on clinical manifestations, the repetitive stimulation test (greater than 10% decrement in amplitude), and the presence of AChR antibodies (AChR antibodies titers > 0.2 nM/L before 2012 or > 0.5 nM/L after 2015) [1, 17, 18]. Clinical information, including medical history, neurological examination, laboratory studies, and treatment responsiveness, were collected (Supplementary Table 2). After manually excluding misdiagnoses and those with missing data, a total of 272 patients were included in the final analysis (Fig. 1).

### Statistical analyses

Statistical analyses were performed using SPSS software 28.0 version (IBM). The normality of the data distribution was tested using the Kolmogorov–Smirnov test or the Shapiro–Wilk test. Quantitative variables were expressed as the median and interquartile range (IQR). Qualitative variables were expressed as percentages. A Pearson Chi-square test with Fisher’s exact test were used for qualitative variables, while Mann–Whitney U tests were used for quantitative variables. To identify risk factors for recurrent infection, we further performed univariate and multivariate logistic regression calculating odds ratios (OR) with 95% confidence intervals (95% CI). The statistical significance was defined as *P-value* < 0.05.

## Results

### Baseline characteristics and clinical features

**Fig. 2:**
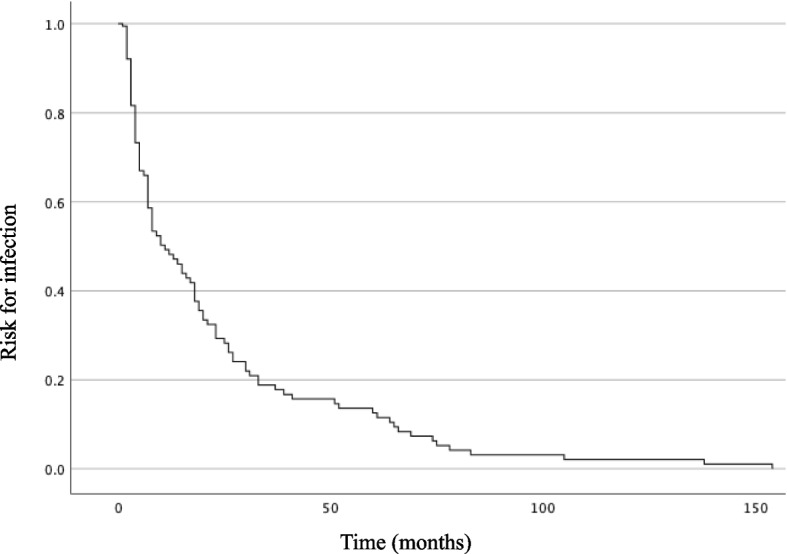
Life table displaying the time (in months) between the first and second infection

Among 272 patients, 176 (64.7%) and 96 (35.3%) patients were classified into the non-recurrent and recurrent groups, respectively. Recurrence happened after a median of 10.24 months, with most happening within the first two months (Fig. 2). Patients in the recurrent group were significantly older than those in the non-recurrent group (median age, 58.5 versus 52.0 years, *p* = 0.024). The non-recurrent group experienced less effects on speech function (76.0% versus 90.3%, *p* = 0.001), while more patients in the recurrent group had endotracheal intubation at the initial hospitalization (17.7% versus 5.7%, *p* = 0.002), lower summation of Medical Research Council scale (MRC) of four sampled muscles (19.5 versus 20.0, *p* = 0.003), diabetes mellitus (22.9% versus 10.2%, *p* = 0.005), deep venous thrombosis (5.2% versus 0.6%, *p* = 0.022), thymic cancer (25.0% versus 13.1%, *p* = 0.013), and prolonged hospitalization (day, 16.5 versus 14.0, *p* = 0.004). A higher proportion of MG patients with recurrent infection were treated with plasmapheresis compared to the non-recurrent group (28.1% versus 17.6%,* p* = 0.043). Furthermore, lower levels of potassium, magnesium, and albumin, and higher serum creatinine level were seen in the recurrent group. However, the percentage of patients with AChR antibody seropositivity was not statistically significant between the non-recurrent and recurrent groups. Hypertension and other cardiovascular diseases, including heart failure, coronary artery disease, and arrhythmia, accounted for the most common comorbidities without statistically different between the two groups (Table 1, Supplementary Table 3).

**Table 1:** Demographic and clinical characteristics upon initial admission between MG patients with and without recurrent infection

	**Total**	**Non-recurrent**	**Recurrent**	***P-value***
**Number of patients**	272/272 (100%)	176/272 (64.7%)	96/272 (35.3%)	NA
**Male**	115/272 (42.3%)	72/176 (40.9%)	43/96 (44.8%)	0.536
**Age, year**	53.0 (39.3–67.9)	52.0 (35.3–66.0)	58.5 (44.0–71.0)	0.024^*^
**Respiratory and bulbar function**				
V5	232/272 (85.3%)	159/176 (90.3%)	73/96 (76.0%)	0.001^**^
VE	27/272 (9.9%)	10/176 (5.7%)	17/96 (17.7%)	0.002^**^
**Motor scale (MRC)**				
Bilateral deltoid and iliopsoas	20.0 (16.0–20.0)	20.0 (18.0–20.0)	19.5 (16.0–20.0)	0.003^**^
**Concomitant diseases**				
Diabetes mellitus	49/272 (14.7%)	18/176 (10.2%)	22/96 (22.9%)	0.005^**^
Deep vein thrombosis	6/272 (2.2%)	1/176 (0.6%)	5/96 (5.2%)	0.022^a*^
Thymic cancer	47/272 (17.3%)	23/176 (13.1%)	24/96 (25.0%)	0.013^*^
**Duration of hospitalization, day**	15.0 (9.0–23.0)	14.0 (8.0–21.0)	16.5 (12.0–28.0)	0.004^**^
**Foley catheterization**	96/272 (35.3%)	69/176 (39.2%)	27/96 (28.1%)	0.068
**Ventilator dependency**	74/272 (27.2%)	51/176 (29.0%)	23/96 (24.0%)	0.374
**Plasmapheresis**	58/272 (21.3%)	31/176 (17.6%)	27/96 (28.1%)	0.043^*^
**Seropositivity of AChR antibody**	215/272 (79.0%)	133/176 (75.6%)	82/96 (85.4%)	0.056
**Laboratory data**				
Hb, g/dL	13.3 (12.2–14.6)	13.6 (12.4–14.9)	13.1 (11.6–14.1)	0.051
Creatinine, mg/dL	0.8 (0.6–1.0)	0.7 (0.6–0.9)	0.8 (0.7–1.0)	0.014^*^
K, mEq/L	3.9 (3.5–4.2)	3.9 (3.6–4.2)	3.8 (3.5–4.1)	0.021^*^
Mg, mEq/L	1.8 (1.6–1.9)	1.8 (1.6–2.0)	1.7 (1.5–1.9)	0.032^b*^
Albumin, g/dL	4.0 (3.3–4.4)	4.1 (3.4–4.4)	3.9 (3.2–4.3)	0.037^*^
APTT, sec	26.5 (23.9–29.2)	26.5 (23.9–28.6)	26.5 (24.3–31.1)	0.158
Cortisol, μg/dL	12.8 (5.4–18.0)	14.0 (8.9–18.5)	9.9 (3.2–17.6)	0.079
**Pneumonia**	49/272 (18.0%)	25/176 (14.2%)	24/96 (25.0%)	0.027^*^
**UTI**	25/272 (9.2%)	11/176 (6.3%)	14/96 (14.6%)	0.023^*^
**Bacteremia**	17/272 (6.3%)	11/176 (6.3%)	6/96 (6.3%)	1.000
**Multiple infections**	18/272 (6.6%)	10/176 (5.7%)	8/96 (8.3%)	0.401

*AChR* acetylcholine receptor, *APTT* activated partial thromboplastin time, *GCS* Glasgow coma scale, *Hb* hemoglobin, *K* potassium, *MG* myasthenia gravis, *Mg* magnesium, *MRC* Medical Research Council scale, *NA* not applicable, *UTI* urinary tract infection, *V5* oriented to verbal response of GCS and no significant respiratory distress and bulbar impairment, *VE* difficulty in evaluating GCS due to endotracheal intubation indicating severe bulbar impairment or respiratory distress. A *p*-value below 0.05 was considered statistically significant. ^*^p < 0.05; ^**^p < 0.01; ^a^Fisher’s exact test; ^b^Two-Sample t test. Unless otherwise reported, qualitative values were expressed as percentage (n/total, %), quantitative values were median (interquartile range IQR)

### Infection types and subgroup analysis

**Fig. 3:**
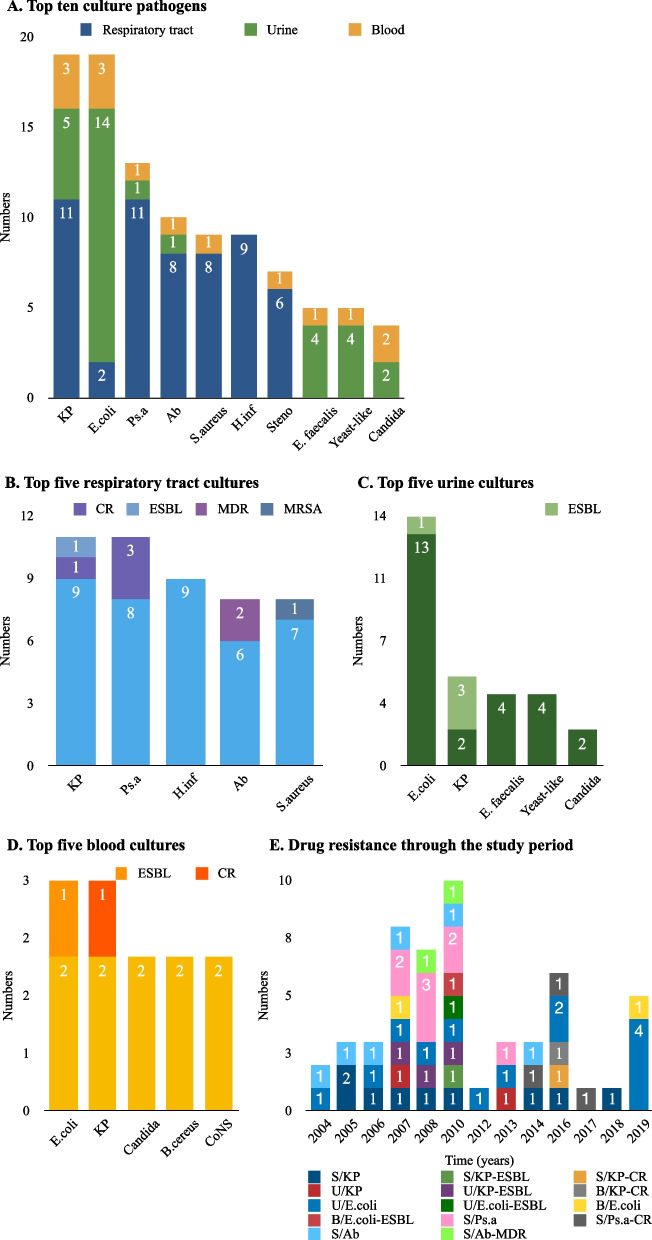
Distribution of cultures and pathogens. **A** shows the top ten common pathogens found in respiratory tract, urine and blood cultures. **B-D** shows the top five most common pathogens found in respiratory tract, urine and blood cultures, separately. **E** shows the tendency of drug resistance through the study period. Ab, *Acinetobacter*; B. cereus, *Bacillus cereus*; B/, Blood cultures; CoNS, Coagulase-negative *staphylococcus*; CR, Carbapenem resistant; E. coli, *Escherichia coli*; E. faecalis, *Enterococcus faecalis*; ESBL, Extended spectrum beta-lactamase; H. inf, *Haemophilus influenzae*; KP, *Klebsiella pneumoniae*; MDR, Multidrug-resistant; MSRA, Methicillin-resistant *Staphylococcus aureus*; Ps. a, *Pseudomonas aeruginosa*; S/, Respiratory tract cultures; S. aureus, *Staphylococcus aureus*; Steno, *Stenotrophomonas maltophilia*; U/, Urine cultures

Pneumonia was the most common infection with a much higher prevalence in the recurrent group (25.0% versus 14.2%, *p* = 0.027). Urinary tract infection was significantly higher in the recurrent group (14.6% versus 6.3%, *p* = 0.023) and was more common than bacteremia. Recurrent patients tended to have multiple infections. However, there was no significant difference in multiple infection rates compared to non-recurrent patients (8.3% versus 5.7%, *p* = 0.401) (Table 1). The top five pathogens were *Klebsiella pneumoniae* (KP), *Escherichia coli* (E. coli), *Pseudomonas aeruginosa* (Ps. a), *Acinetobacter* (Ab), and *Staphylococcus aureus* (S. aureus). KP were the most common pneumonia pathogens and the second most common bacteria in urine and blood cultures. E. coli were the most commonly found bacteria in both urine and blood cultures (Fig. 3A-D). Carbapenem resistant (CR) KP and Ps. a infection rates increased annually since 2014, while extended spectrum beta-lactamase (ESBL) KP, E. coli, and multidrug-resistant (MDR) Ab peaked from 2007 to 2010 (Fig. 3E).

### Predictive factors for recurrent infection

Univariate analysis revealed that the status post endotracheal intubation at MC admission predicted the risk of recurrent infection (OR 3.57; 95% CI 1.56–8.16; *p* = 0.003). In contrast, the use of a mechanical ventilator and a poor weaning profile throughout the course of hospitalization predicted a lower risk of infection, but without a statistical difference (OR 0.77; 95% CI 0.44–1.37; *p* = 0.375). Intact bulbar function was associated with a lower risk of recurrent infection than full proximal limb muscle strength (OR 0.34; 95% CI 0.17–0.67; *p* = 0.002; versus OR 0.94; 95% CI 0.88–1.01; *p* = 0.101). In addition, old age, diabetes mellitus, deep venous thrombosis, thymic cancer, longer length of hospital stay, plasmapheresis, lower levels of hemoglobin, potassium, magnesium and albumin, and prolonged aPTT were associated with recurrent infection. After adjustment, only diabetes mellitus (OR 83.11; 95% CI 1.25–5512.32; *p* = 0.039), length of hospital stays (OR 1.14; 95% CI 1.02–1.26; *p* = 0.015), levels of magnesium (OR 0.01; 95% CI 0.00–0.54; *p* = 0.024) and aPTT (OR 1.96; 95% CI 1.03–3.70; *p* = 0.038) were associated with recurrence. Though not statistically significant, we observed an inverse association between cortisol levels, Foley catheterization, and infection. Detailed information is presented in Table 2 and Supplementary Table 4.

**Table 2:** Univariate and multivariate analysis via binary logistic regression of variables associated with recurrent infection of MG

	**Crude OR** **[95% CI]**	**Adjusted OR** **[95% CI]**	***P-value***	**Adjusted** ***P-value***
**Male**	1.17 [0.71–1.94]	-	0.536	-
**Age**	1.02 [1.00–1.03]	0.93 [0.87–1.01]	0.028^*^	0.101
**Respiratory and bulbar function**				
V5	0.34 [0.17–0.67]	0.34 [0.01–15.48]	0.002^**^	0.582
VE	3.57 [1.56–8.16]	0.16 [0.00–9.54]	0.003^**^	0.379
**Motor scale (MRC)**				
Bil. deltoid and iliopsoas	0.94 [0.88–1.01]	-	0.101	-
**Concomitant diseases**				
Diabetes mellitus	2.61 [1.32–5.16]	83.11 [1.25–5512.32]	0.006^**^	0.039^*^
Deep vein thrombosis	9.62 [1.11–83.5]	4.75 [0.05–464.08]	0.040^*^	0.505
Thymic cancer	2.22 [1.17–4.19]	1.67 [0.12–22.99]	0.014^*^	0.702
**Duration of hospitalization**	1.03 [1.01–1.05]	1.14 [1.02–1.26]	0.006^**^	0.015^*^
**Foley catheterization**	0.61 [0.34–1.04]	-	0.069	-
**Ventilator dependency**	0.77 [0.44–1.37]	-	0.375	-
**Plasmapheresis**	1.83 [1.01–3.30]	0.33 [0.03–4.00]	0.045^*^	0.386
**AChR-antibody positive**	1.89 [0.98–3.68]	-	0.059	-
**Laboratory data**				
Hb	0.88 [0.78–1.00]	1.04 [0.56–1.94]	0.037^*^	0.891
Creatinine	1.08 [0.90–1.30]	-	0.385	-
K	0.49 [0.28–0.85]	0.22 [0.01–2.43]	0.011^*^	0.215
Mg	0.16 [0.03–0.89]	0.01 [0.00–0.54]	0.037^*^	0.024^*^
Albumin	0.63 [0.41–0.96]	0.47 [0.07–3.26]	0.030^*^	0.449
APTT	1.08 [1.01–1.16]	1.96 [1.03–3.70]	0.026^*^	0.038^*^
Cortisol	0.96 [0.91–1.00]	-	0.065	-

*AChR* acetylcholine receptor, *APTT* activated partial thromboplastin time, *Bil.* Bilateral, *GCS* Glasgow coma scale, *Hb* hemoglobin, *K* potassium, *MG* myasthenia gravis, *Mg* magnesium, *MRC* Medical Research Council scale, *V5* oriented to verbal response of GCS and no significant respiratory distress and bulbar impairment, *VE* difficulty in evaluating GCS due to endotracheal intubation indicating severe bulbar impairment or respiratory distress. Risk for recurrent infection in MG patients was presented as odds ratio (OR). 95% CI, 95% confidence interval. A *p*-value below 0.05 was considered statistically significant. ^*^p < 0.05; ^**^p < 0.01

## Discussion

We found MG patients with bulbar weakness or respiratory distress, rather than limb weakness, at initial hospitalization to be at higher risk of recurrent infection. This is similar to Thomas et al., who found that limb involvement could be absent in 20% of MC, meaning that the respiratory crisis was usually, but not always, associated with GMG [7]. The timing of the respiratory salvage intervention predicted the risk of recurrence. Patients with lower vital capacities, less than 15 ml/kg, before and during admission, which generally indicated intubation and the need for a ventilator, had poorer outcomes [7, 9]. Patients who had a higher MGFA class before MC and at admission, prolonged intubation, or a longer total hospital stay had a higher likelihood of functional dependence at discharge as well [7, 9]. We assumed patients who experienced respiratory failure immediately at admission either had prominent bulbar muscle weakness as their baseline condition or had an immunocompromising condition, which resulted in vulnerability to infection and a high demand for airway security. On the other hand, patients who were intubated during hospitalization, except for emergent conditions, such as a scheduled operation, avoided prolonged intubation and hospital stays, which reduced the prevalence of complications such as pneumonia and anemia [7, 9, 19].

Myasthenia gravis is well known to be associated with comorbidities, and the number of them at admission was related to worse outcomes [9]. Hypertension and other cardiovascular diseases accounted for the most common category. Pulmonary diseases represented a smaller portion: 16% to 33% in previous studies and around 3% in our cohort [7, 9, 20]. Both cardiovascular and pulmonary diseases were associated with MC and exacerbation, but we did not find the similar finding or increased heart disease mortality compared to the general population, although thymoma-related cardiac as well as lung tissue invasion had been proposed [3, 9, 20–22].

Consistent with previous studies, patients with thymic cancer tended to have more frequent infections, especially of the respiratory tract, contributing to an increased level of inflammatory cytokines [22, 23]. About 10% to 15% of MG patients had thymoma, particular in those seropositivity group of AChR antibodies [1, 4, 8, 22]. Thymoma-induced immune dysregulation and loss of self-tolerance not only provided a primary source of AChR antibodies but also reduced the systemic defenses against pathogens [3, 6, 22]. Microbes are thought to precipitate an unwanted cross-reactivity for T-cells and B-cells by means of molecular mimicry and local inflammation of the target organ, leading to increased immunogenicity of self-antigens and polyclonal activation of B and T lymphocytes, which then results in the onset of MG [4, 22, 24]. Moreover, immunosuppressive therapy, which most GMG patients receive, suppresses immune reactivity against microbial antigens, and plasmapheresis reduces the concentration of cytokines as well as protective antibodies in the same way as pathogenic autoantibodies [12, 22, 25].

Infection has been suggested as the major causal factor of the patients with autoimmune disorders for concordant MG [9, 26, 27]. Diabetes mellitus accounts for 14.7% of all infections in MG patients and serves as the most reliable predictive value, regardless of HbA1c or blood sugar levels at admission. Neither HbA1c nor blood sugar levels were associated with MC intubation [7]. Up to 10% of MG patients have autoimmune thyroid conditions, but it is unclear whether changes in thyroid status were linked to MG exacerbations or not [1, 8–10, 21]. In our study, neither hyperthyroidism nor hypothyroidism predicted infection. Even cortisol level was not associated with MC, although the risk for infection while on corticosteroid therapy could be increased by 50%. In particular, females are twice as likely as males to develop transient exacerbations and MC within the first 2 weeks [9, 22, 28, 29].

Using univariate analysis, we found deep vein thrombosis and plasmapheresis during hospitalization to be associated with recurrent infection. While there have been advances in the management of both AChR-antibody positive and MuSK-antibody positive patients, plasmapheresis remains the first-line treatment for MC [1, 8, 30–32]. However, its use increases the risk of thrombotic complications, catheter-related infection, and even systemic infection, especially for those who delayed therapy for more than two days from admission [1, 2, 30, 31, 33, 34]. Other adverse effects included bleeding, hemolysis, decreased fibrinogen that required fresh frozen plasma transfusion, hypersensitivity to albumin, cardiac complications, and acute renal failure, which presented as elevated blood urea nitrogen (BUN) and hypokalemia [1, 2, 31, 33]. Certain characteristics were statistically different between the non-recurrent and recurrent infection groups. The latter were more likely to present with elevated BUN and creatine, electrolyte imbalance, hypoalbuminemia, and coagulopathy. We must be alert to this to prevent patients from getting trapped in this vicious cycle of infection, MC, plasmapheresis, and its associated side effects. Subcutaneous heparinization, pneumatic compression devices, and elastic stockings were critical in the prevention of deep vein thrombosis in GMG patients [35]. Peripheral rather than central venous access could lower the rates of deep vein thrombosis, coagulopathy, and anemia [31]. Use of a different brand of albumin supplement may help get rid of anaphylactic reactions, but one should keep in mind that it is the chronic inflammation that is responsible for morbidity and mortality [23, 33, 36]. Instead of merely correcting low albumin levels, it is crucial to treat underlying autoimmune diseases. Among the electrolyte disturbances that contribute to muscle weakness and possible respiratory failure, we discovered that potassium and magnesium deficiency were associated with infection. In critically ill patients, hypomagnesemia is common and significantly associated with older age, lower albumin, potassium, calcium and phosphate, and anemia [37–40]. A deficiency of magnesium intake with malnutrition has been reported to increase the risk for infection-related mortality, while the possibility of reverse causality should be taken in mind [40, 41]. Physicians should closely monitor and regularly assess the development of any MG symptoms when treating symptomatic hypomagnesaemia, which might in turn induce a MC episode [37, 42]. Meanwhile, improving general nutritional status rather than pure magnesium supplementation may be more beneficial [41].

Antibiotics are a double-edged sword for patients with MG because infection, particularly bacterial pneumonia, triggers MC, which is the topic we are interested in [1, 7, 20]. Fluoroquinolone exposure, a common indication for respiratory or urinary tract infection, has been shown to prolong hospitalization, and aminoglycosides have been implicated in causing intensive care unit (ICU)-related weakness. Both should be avoided because of their ability to induce MC [13, 42, 43]. Macrolides, lactams, tetracyclines, and quinines have also been linked to an increase in the risk of MC [1, 42, 43].

Urinary catheterization is known to increase the risk of complicated urinary tract infection, but our analysis found opposite results [44, 45]. The indication for catheter usage was mainly during thymectomy and was removed early post-operation, thereby minimizing infection risk [44, 46]. Others who were catheterized were critically ill patients. In this case, urinary tract infection appeared because of prolonged intubation and immobilization, rather than as the primary cause of MC [7, 35, 44, 46]. Pelvic muscle weakness led to urinary incontinence without the need for indwelling catheters, but it also predisposed patients to urinary tract infections [22, 47].

We were not surprised to find there was no association between infection and white blood cell (WBC) count or C-reactive protein (CRP). In the setting of MG, in addition to the systemic inflammatory status with CD4 + T cells and cytokines that contribute to the development of the disease itself, cytokines are known to influence the acute phase of protein production [6, 45]. Being nonspecific for the acute phase of an infectious or inflammatory condition, single levels of WBC and CRP are not reliable diagnostic indicators; instead, they are useful in evaluating the disease’s time course, complications, or antibiotic treatment responses in patients with pneumonia and urinary tract infection [45, 48, 49]. Also, we didn’t find a correlation between AChR antibodies and infection. Whether AChR seropositive or negative, there was a risk of conversion to GMG, but it wasn’t associated with disease severity [1, 3, 26, 50].

The single-center retrospective design of our study had several limitations. Data were collected during daily clinical practice rather than in a formal study setting, which led to variation in both quantity and quality between individuals. We failed to carry out an objective efficacy scale to evaluate the disease’s endpoints [1, 31, 51]. As a result of the above description and medical charts, muscle strength may be overestimated or underestimated. Recording only at a single time point might be insufficient to cover the dynamic essentials. Also, people of Asian ancestry have relatively higher rates of MuSK antibodies, which leads to a higher risk of GMG and MC compared to the AChR seropositive group [1, 3, 5, 6, 26]. However, we didn’t recruit for that in this study due to a lack of serologic testing confirmation, though those that had been studied to be a problem for MG patients. Immunosuppressive treatment also increases the risk of infection and could be a confounder [20]. Nevertheless, medication records involving steroids and immunosuppressants were excluded from this study to ensure that the interpretation of the analysis results is not confounded by the complexities associated with frequent dosage adjustments, the timing of adjunct therapies, and the management of pre-existing comorbidities.

We only recorded whether patients had concomitant thymic cancer, regardless of whether they underwent a thymectomy or not. Thymectomy is widely advocated due to its long-term beneficial effects, like decreasing AChR antibody titers, minimizing immunotherapy requirements, reducing hospitalization due to MG exacerbation, but peri-operative complications that reduce respiratory or circulatory function may occur [3, 8, 21, 27, 29, 34]. To date, there is scarce evidence comparing infection frequency and severity in MG patients before and after thymectomy, although the immunosuppressive effect of thymectomy may increase vulnerability to infection [22, 27]. Finally, milder infection in MG might be underestimated because we limited our study to infection requiring hospitalization. Conversely, prolonged prophylactic use of antibiotics for elective surgery might lead to an overestimation [52, 53].

Despite these limitations, the risk factors identified in this study offered practical and feasible insights that can be implemented to assist in the development of targeted interventions aimed at preventing recurrent infections patients with MG. Additionally, our finding on the presentation of infectious pathogens and cultures may be helpful in determining appropriate medical treatment. Further research and prospective studies are necessary to validate these findings and refine interventions in order to optimize patient care and improve clinical outcomes.

## Conclusions

Having diabetes and prolonged hospitalization are independent risk factors for recurrent infection in patients with MG. Patients who present with bulbar muscle weakness or respiratory distress as their baseline are at high risk of infection. Electrolyte imbalances at admission, especially hypomagnesaemia, make MG patients vulnerable to infection as well, but they should be treated cautiously to avoid drug-induced MC. Prophylaxis and correction of deep vein thrombosis, coagulopathy, anemia, and hypoalbuminemia in patients who undergo plasmapheresis during hospitalization may be reasonable to prevent recurrent infection. While thymic cancer plays an important role in chronic inflammation as well as immune dysregulation, peri-thymectomy indwelling catheters should be removed early to minimize the risk of infection. In summary, this study has contributed to a more comprehensive perspective on the management of recurrent infection in patients with MG. The intervention targets identified for recurrent infections in this study provide valuable insights that may refine the approach to patient care.

## Supplementary Information

**Additional file 1.** Identified myasthenia gravis inpatients based on the corresponding International Classification of Diseases (ICD) code, Ninth or Tenth Edition.

**Additional file 2.** Detailed items collected for each patient upon first admission in the study.

**Additional file 3.** Demographic and clinical characteristics upon initial admission between MG patients with and without recurrent infection (full version).

**Additional file 4.** Univariate analysis via binary logistic regression of variables associated with recurrent infection of MG (full version).

## Data Availability

The datasets used and/or analysed during the current study are available from the corresponding author on reasonable request.

## Abbreviations

Ab: *Acinetobacter*
AChR: Acetylcholine receptor
ALP: Alkaline phosphatase
ANA: Antinuclear antibody
aPTT: Activated partial thromboplastin time
AST: Aspartate aminotransferase
B/: Blood cultures
B. cereus: *Bacillus cereus*
Bil: Bilateral
BUN: Blood urea nitrogen
Ca: Calcium
CI: Confidence interval
CK: Creatine kinase
CL: Chloride
CR: Carbapenem resistant
CoNS: Coagulase-negative *staphylococcus*
CRP: C-reactive protein
E. coli: *Escherichia coli*
E. faecalis: *Enterococcus faecalis*
ESBL: Extended spectrum beta-lactamase
Fe: Iron
Free T4: Free thyroxine
GCS: Glasgow coma scale
GMG: Generalized myasthenia gravis
Hb: Hemoglobin
HbA1c: Glycohemoglobin
Hct: Hematocrit
HDL: High density lipoprotein
H. inf: *Hemophilus influenza*
ICU: Intensive care unit
IQR: Interquartile range
K: Potassium
KP: *Klebsiella pneumoniae*
LDH: Lactate dehydrogenase
LDL: Low density lipoprotein
MC: Myasthenic crisis
MCH: Mean corpuscular hemoglobin
MCHC: Mean corpuscular hemoglobin concentration
MCV: Mean corpuscular volume
MDR: Multidrug-resistant
Mg: Magnesium
MG: Myasthenia gravis
MGFA: Myasthenia Gravis Foundation of America
MSRA: Methicillin-resistant *Staphylococcus aureus*
MuSK: Muscle specific kinase
MRC: Medical Research Council scale
NA: Not applicable
Na: Sodium
OMG: Ocular myasthenia gravis
OR: Odds ratio
P: Phosphorus
Ps.A: *Pseudomonas aeruginosa*
PT/INR: Prothrombin ratio/international normalized ratio
RBC: Red blood cell
RDW: Red cell distribution width
r-GT: R-glutamyltransferase
S/: Respiratory tract cultures
S. aureus: *Staphylococcus aureus*
Steno: *Stenotrophomonas maltophilia*
T3: Triiodothyronine
T4: Thyroxine
TG: Triglyceride
TIBC: Total iron binding capacity
TSH: Thyroid-stimulating hormone
U/: Urine cultures
UA: Uric acid
UTI: Urinary tract infection
V5: Oriented to verbal response of GCS and no significant respiratory distress and bulbar impairment
VE: Difficulty in evaluating GCS due to endotracheal intubation indicating severe bulbar impairment or respiratory distress
VLDL: Very low density lipoprotein
VT: Difficulty in evaluating GCS due to tracheostomy indicating severe bulbar impairment or respiratory distress
WBC: White blood cell
