# Supplementary material for: Myasthenia gravis and independent risk factors for recurrent infection: a retrospective cohort study
Source: BMC Neurol. 2023 Jul 3;23:255. doi: 10.1186/s12883-023-03306-3 (PMC10316583; doi:10.1186/s12883-023-03306-3)
Supplement: Supplementary file 1 — Additional file 1. Identified myasthenia gravis inpatients based on the corresponding International Classification of Diseases (ICD) code, Ninth or Tenth Edition. [file 12883_2023_3306_MOESM1_ESM.pdf]

Supplementary Table 1. Identified myasthenia gravis inpatients based on the corresponding International Classification of Diseases (ICD) code, Ninth or Tenth Edition.

**Code**	
**ICD-9**	
3580	Myasthenia gravis
3581	Myasthenic syndrome in disease classified elsewhere
3582	Toxic myoneural disorders
3588	Other specified myoneural disorders
3589	Myoneural disorders, unspecified
**ICD-10**	
G7000	Myasthenia gravis without (acute) exacerbation
G7001	Myasthenia gravis with (acute) exacerbation
G709	Myoneural disorder, unspecified
G733	Myasthenic syndrome in other disease classified elsewhere
