# Supplementary material for: Myasthenia gravis and independent risk factors for recurrent infection: a retrospective cohort study
Source: BMC Neurol. 2023 Jul 3;23:255. doi: 10.1186/s12883-023-03306-3 (PMC10316583; doi:10.1186/s12883-023-03306-3)
Supplement: Supplementary file 2 — Additional file 2. Detailed items collected for each patient upon first admission in the study. [file 12883_2023_3306_MOESM2_ESM.pdf]

Supplementary Table 2. Detailed items collected for each patient upon first admission in the study.

**Gender**
**Age**
**Respiratory and bulbar function (verbal response of Glasgow coma scale)**
**Motor scale (Medical Research Council scale)**
**Concomitant diseases**
**Duration of hospitalization (admission and discharge date)**
**Foley catheterization**
**Ventilator dependency**
**Nasogastric tube**
**Central venous catheter**
**Plasmapheresis**
**Acetylcholine receptor (AChR) antibody status**
**Laboratory data**
Complete blood count with differential
Renal function and electrolytes
Hepatobiliary function
Albumin
Coagulative function
Lipid profile
Inflammatory and autoimmune biomarkers
Glycohemoglobin (HbA1c)
Thyroid function
Cortisol
**Cultures**
Sputum
Urine
Blood
