# Supplementary material for: Myasthenia gravis and independent risk factors for recurrent infection: a retrospective cohort study
Source: BMC Neurol. 2023 Jul 3;23:255. doi: 10.1186/s12883-023-03306-3 (PMC10316583; doi:10.1186/s12883-023-03306-3)
Supplement: Supplementary file 3 — Additional file 3. Demographic and clinical characteristics upon initial admission between MG patients with and without recurrent infection (full version). [file 12883_2023_3306_MOESM3_ESM.pdf]

Supplementary Table 3. Demographic and clinical characteristics upon initial admission between MG patients with and without recurrent infection (full version).

	**Total**	**Non-recurrent**	**Recurrent**	***P-value***
**Number of patients**	272/272 (100%)	176/272 (64.7%)	96/272 (35.3%)	NA
**Male**	115/272 (42.3%)	72/176 (40.9%)	43/96 (44.8%)	0.536
**Age, year**	53.0 (39.3-67.9)	52.0 (35.3-66.0)	58.5 (44.0-71.0)	0.024*
**Respiratory and bulbar function**				
V5	232/272 (85.3%)	159/176 (90.3%)	73/96 (76.0%)	0.001**
V4	6/272 (2.2%)	4/176 (2.3%)	2/96 (2.1%)	1.000[a]
V3	0/272 (0.0%)	0/176 (0.0%)	0/96 (0.0%)	NA
V2	1/272 (0.4%)	1/176 (0.6%)	0/96 (0.0%)	1.000[a]
V1	3/272 (1.1%)	0/176 (0.0%)	3/96 (3.1%)	0.043[a]*
VA	1/272 (0.4%)	1/176 (0.6)	0/96 (0.0%)	1.000[a]
VE	27/272 (9.9%)	10/176 (5.7%)	17/96 (17.7%)	0.002**
VT	2/272 (0.7%)	1/176 (0.6%)	1/96 (1.0%)	1.000[a]
**Motor scale (MRC)**				
Right deltoid	5.00 (4.00-5.00)	5.00 (4.25-5.00)	5.00 (4.00-5.00)	0.018*
Left deltoid	5.00 (4.00-5.00)	5.00 (4.00-5.00)	5.00 (4.00-5.00)	0.017*
Right iliopsoas	5.00 (4.00-5.00)	5.00 (5.00-5.00)	5.00 (4.00-5.00)	0.005**
Left iliopsoas	5.00 (4.00-5.00)	5.00 (4.25-5.00)	5.00 (4.00-5.00)	0.004**
Right deltoid and iliopsoas	10.00 (8.00-10.00)	10.00 (9.00-10.00)	10.00 (8.00-10.00)	0.005**
Left deltoid and iliopsoas	10.00 (8.00-10.00)	10.00 (9.00-10.00)	10.00 (8.00-10.00)	0.007**
Bilateral deltoid	10.00 (8.00-10.00)	10.00 (9.00-10.00)	10.00 (8.00-10.00)	0.009**
Bilateral iliopsoas	10.00 (8.00-10.00)	10.00 (9.00-10.00)	10.00 (8.00-10.00)	0.002**
Bilateral deltoid and iliopsoas	20.00 (16.00-20.00)	20.00 (18.00-20.00)	19.50 (16.00-20.00)	0.003**
**Concomitant diseases**				
Hypertension	72/272 (26.5%)	44/176 (25.0%)	28/96 (29.2%)	0.457

	Total	Non-recurrent	Recurrent	*P-value*
Diabetes mellitus	49/272 (14.7%)	18/176 (10.2%)	22/96 (22.9%)	0.005**
Dyslipidemia	13/272 (4.8%)	8/176 (4.5%)	5/96 (5.2%)	0.775[a]
Congestive heart failure	9/272 (3.3%)	7/176 (4.0%)	2/96 (2.1%)	0.500[a]
Coronary artery disease	7/272 (2.6%)	5/176 (2.8%)	2/96 (2.1%)	1.000[a]
Atrial fibrillation	6/272 (2.2%)	4/176 (2.3%)	2/96 (2.1%)	1.000[a]
Deep vein thrombosis	6/272 (2.2%)	1/176 (0.6%)	5/96 (5.2%)	0.022[a]*
Asthma	5/272 (1.8%)	2/176 (1.1%)	3/96 (3.1%)	0.349[a]
Chronic obstructive pulmonary disease	3/272 (1.1%)	2/176 (1.1%)	1/96 (1.0%)	1.000[a]
Interstitial lung disease	1/272 (0.4%)	0/176 (0.0%)	1/96 (1.0%)	0.353[a]
Gastroesophageal reflux disease	7/272 (2.6%)	5/176 (2.8%)	2/96 (2.1%)	1.000[a]
Peptic ulcer disease	16/272 (5.9%)	8/176 (4.5%)	8/96 (8.3%)	0.205
Hepatitis B	5/272 (1.8%)	4/176 (2.3%)	1/96 (1.0%)	0.659[a]
Hepatitis C	3/272 (1.1%)	3/176 (1.7%)	0/96 (0.0%)	0.554[a]
Liver cirrhosis	6/272 (2.2%)	3/176 (1.7%)	3/96 (3.1%)	0.669[a]
Acute kidney injury	3/272 (1.1%)	2/176 (1.1%)	1/96 (1.0%)	1.000[a]
Chronic kidney disease	7/272 (2.6%)	5/176 (2.8%)	2/96 (2.1%)	1.000[a]
End stage renal disease	1/272 (0.4%)	1/176 (0.6%)	0/96 (0.0%)	1.000[a]
Benign Prostatic Hyperplasia	6/272 (2.2%)	4/176 (2.3%)	2/96 (2.1%)	1.000[a]
Hyperthyroidism	14/272 (5.1%)	11/176 (6.3%)	3/96 (3.1%)	0.391[a]
Hypothyroidism	3/272 (1.1%)	1/176 (0.6%)	2/96 (2.1%)	0.285[a]
Cushing syndrome	4/272 (1.5%)	3/176 (1.7%)	1/96 (1.0%)	1.000[a]
Addison’s disease	7/272 (2.6%)	2/176 (1.1%)	5/96 (5.2%)	0.101[a]
Gout	5/272 (1.8%)	4/176 (2.3%)	1/96 (1.0%)	0.659[a]
Myositis	3/272 (1.1%)	2/176 (1.1%)	1/96 (1.0%)	1.000[a]
Systemic lupus erythematosus	5/272 (1.8%)	3/176 (1.7%)	2/96 (2.1%)	1.000[a]

	Total	Non-recurrent	Recurrent	*P-value*
Iron deficiency anemia	6/272 (2.2%)	2/176 (1.1%)	4/96 (4.2%)	0.189[a]
Thymic cancer	47/272 (17.3%)	23/176 (13.1%)	24/96 (25.0%)	0.013*
**Duration of hospitalization, day**	15.0 (9.0-23.0)	14.0 (8.0-21.0)	16.5 (12.0-28.0)	0.004**
**Foley catheterization**	96/272 (35.3%)	69/176 (39.2%)	27/96 (28.1%)	0.068
**Ventilator dependency**	74/272 (27.2%)	51/176 (29.0%)	23/96 (24.0%)	0.374
**Nasogastric tube**	82/272 (30.1%)	55/176 (31.3%)	27/96 (28.1%)	0.591
**Central venous catheter**	63/272 (23.2%)	46/176 (26.1%)	17/96 (17.7%)	0.115
**Plasmapheresis**	58/272 (21.3%)	31/176 (17.6%)	27/96 (28.1%)	0.043*
**Seropositivity of AChR antibody**	215/272 (79.0%)	133/176 (75.6%)	82/96 (85.4%)	0.056
**Laboratory data**				
WBC, 1000/uL	8.80 (6.80-11.90)	8.40 (6.80-11.45)	9.70 (6.60-12.90)	0.227
Neutrocyte, %	73.00 (60.85-83.40)	72.00 (60.00-82.00)	74.50 (63.43-86.00)	0.084
Lymphocyte, %	18.00 (10.85-29.40)	19.10 (12.00-31.00)	16.95 (8.08-26.25)	0.061
Monocyte, %	5.10 (3.80-6.90)	5.10 (3.90-6.70)	5.10 (3.00-7.00)	0.651
Eosinophil, %	1.00 (0.10-2.00)	1.00 (0.20-2.00)	1.00 (0.10-1.85)	0.308
Basophil, %	0.20 (0.10-0.40)	0.20 (0.10-0.50)	0.20 (0.10-0.40)	0.381
RBC, million/uL	4.55 (4.20-4.97)	4.61 (4.24-5.02)	4.49 (4.12-4.91)	0.106
Hct, %	40.10 (36.80-43.40)	40.60 (37.33-43.50)	39.30 (35.30-43.20)	0.097
Hb, g/dL	13.30 (12.20-14.60)	13.55 (12.43-14.88)	13.10 (11.60-14.10)	0.051
MCH, pg/Cell	29.70 (28.30-30.80)	29.70 (28.50-30.68)	29.80 (28.10-30.80)	0.772
MCHC, gHb/dL	33.30 (32.50-33.90)	33.30 (32.60-34.00)	33.00 (32.30-33.80)	0.142
MCV, fL	88.90 (85.30-91.90)	88.85 (85.30-91.48)	89.00 (85.20-93.10)	0.500
RDW, %	13.20 (12.80-14.30)	13.20 (12.70-14.20)	13.30 (12.80-14.90)	0.194
Platelet, 1000/uL	237.00 (193.00-283.00)	239.00 (198.00-288.00)	232.00 (177.00-270.00)	0.104
BUN, mg/dL	13.95 (10.18-18.00)	13.00 (10.43-17.00)	14.15 (10.00-18.15)	0.373

	Total	Non-recurrent	Recurrent	*P-value*
Creatinine, mg/dL	0.75 (0.60-1.00)	0.73 (0.59-0.92)	0.80 (0.65-1.04)	0.014*
Na, mEq/L	140.00 (138.00-142.00)	140.00 (138.00-142.00)	140.00 (138.00-141.75)	0.233
K, mEq/L	3.90 (3.50-4.20)	3.90 (3.60-4.20)	3.80 (3.50-4.10)	0.021*
Ca, mg/dL	8.80 (8.40-9.10)	8.90 (8.40-9.20)	8.70 (8.18-9.10)	0.085
P, mg/dL	3.50 (3.00-4.10)	3.60 (3.10-4.10)	3.35 (2.70-4.30)	0.290
Mg, mEq/L	1.80 (1.60-1.90)	1.80 (1.60-2.00)	1.70 (1.50-1.85)	0.032[b]*
CL, mEq/L	104.50 (102.00-108.00)	104.00 (101.00-107.25)	105.50 (102.75-108.00)	0.114[b]
AST, U/L	22.00 (17.00-29.00)	22.00 (17.25-29.75)	22.00 (16.00-27.00)	0.598
Bilirubin total, mg/dL	0.60 (0.40-0.73)	0.60 (0.40-0.78)	0.60 (0.40-0.73)	0.917
Bilirubin direct, mg/dL	0.30. (0.20-0.40)	0.30. (0.20-0.40)	0.30. (0.20-0.55)	0.320
ALP, U/L	63.00 (50.25-82.50)	63.00 (49.75-80.75)	65.00 (51.75-84.50)	0.665
r-GT, U/L	57.50 (27.50-99.00)	57.00 (24.00-87.00)	59.00 (28.50-152.50)	0.618
Albumin, g/dL	4.00 (3.30-4.40)	4.10 (3.41-4.42)	3.89 (3.19-4.30)	0.037*
PT/INR	1.00 (1.00-1.10)	1.00 (1.00-1.10)	1.10 (1.00-1.10)	0.114
APTT, sec	26.50 (23.88-29.23)	26.50 (23.85-28.55)	26.50 (24.25-31.10)	0.158
D-dimer, FEU ng/mL	1252.00 (499.50-2629.00)	1653.83 (499.50-3323.00)	1225.00 (443.50-1675.00)	0.521
TG, mg/dL	119.00 (85.00-172.00)	119.00 (85.00-171.00)	116.50 (83.75-175.75)	0.968
Cholesterol, mg/dL	179.00 (161.00-206.00)	179.00 (161.00-206.00)	180.50 (156.50-202.00)	0.936
LDL, mg/dL	107.00 (92.50-132.75)	103.50 (88.00-135.25)	108.00 (95.50-127.50)	0.403
VLDL, mg/dL	25.50 (16.00-34.75)	25.00 (15.00-42.00)	28.00 (16.50-34.50)	0.878
HDL, mg/dL	47.00 (37.00-55.00)	47.50 (36.75-54.25)	45.00 (36.50-58.00)	0.346[b]
Lupus anticoagulant	14.60 (9.00-19.90)	14.60 (8.70-19.90)	12.35 (9.00-21.98)	0.930
CK, U/L	64.00 (37.50-133.00)	71.00 (40.00-131.00)	60.50 (36.25-134.00)	0.618
LDH, U/L	214.00 (158.00-422.00)	242.00 (142.00-480.00)	214.00 (179.75-347.25)	0.580
CRP, mg/L	12.14 (2.16-50.30)	12.60 (2.15-54.83)	11.80 (2.09-45.20)	0.914

	Total	Non-recurrent	Recurrent	*P-value*
ANA	0.00 (0.00-0.00)	0.00 (0.00-0.00)	0.00 (0.00-0.00)	0.901
C3, mg/dL	102.00 (84.95-115.00)	103.50 (87.13-124.25)	100.00 (84.25-109.00)	0.219[b]
C4, mg/dL	22.30 (16.95-27.30)	22.00 (17.08-29.45)	22.50 (16.90-25.75)	0.768
Fe, µg/dL	48.00 (25.75-64.75)	41.00 (18.25-105.75)	48.00 (26.50-64.75)	0.633[b]
Ferritin, ng/mL	78.70 (15.90-220.50)	15.90 (6.70-541.25)	84.95 (33.95-151.05)	0.806
TIBC, µg/dL	280.50 (209.50-326.00)	307.50 (202.75-383.75)	276.00 (195.50-320.00)	0.647[b]
HbA1c, %	6.40 (5.70-7.45)	6.20 (5.70-7.00)	6.50 (5.95-7.90)	0.178
Glucose, mg/dL	105.00 (91.00-135.25)	105.00 (92.00-135.75)	106.00 (90.00-135.25)	0.829
TSH, µIU/mL	1.26 (0.65-2.06)	1.15 (0.69-1.94)	1.37 (0.61-2.27)	0.395
Free T4, ng/dL	1.18 (1.04-1.34)	1.19 (1.06-1.35)	1.18 (1.02-1.32)	0.537
T3, ng/dL	95.35 (72.83-115.57)	96.60 (72.85-117.64)	93.90 (62.10-114.60)	0.906[b]
T4, µg/dL	7.90 (6.75-9.58)	7.90 (6.90-9.55)	7.40 (5.40-10.10)	0.661[b]
Cortisol, µg/dL	12.80 (5.40-18.00)	14.00 (8.85-18.5)	9.94 (3.20-17.56)	0.079
UA, mg/dL	5.40 (4.55-6.50)	5.40 (4.38-6.63)	5.30 (4.60-6.40)	0.795
**Pneumonia**	49/272 (18.0%)	25/176 (14.2%)	24/96 (25.0%)	0.027*
**UTI**	25/272 (9.2%)	11/176 (6.3%)	14/96 (14.6%)	0.023*
**Bacteremia**	17/272 (6.3%)	11/176 (6.3%)	6/96 (6.3%)	1.000
**Multiple infections**	18/272 (6.6%)	10/176 (5.7%)	8/96 (8.3%)	0.401

AChR, acetylcholine receptor; ALP, alkaline phosphatase; ANA, antinuclear antibody; APTT, activated partial thromboplastin time; AST, aspartate aminotransferase; BUN, blood urea nitrogen; Ca, calcium; CK, creatine kinase; CL, chloride; CRP, C-reactive protein; Fe, iron; Free T4, free thyroxine; GCS, Glasgow coma scale; Hb, hemoglobin; HbA1c, glycohemoglobin; Hct, hematocrit; HDL, high density lipoprotein; K, potassium; LDH, lactate dehydrogenase; LDL, low density lipoprotein; MCH, mean corpuscular hemoglobin; MCHC, mean corpuscular hemoglobin concentration; MCV, mean corpuscular volume; MG, myasthenia gravis; Mg, magnesium; MRC, Medical Research Council scale; NA, not applicable; Na, sodium; P, phosphorus; PT/INR, prothrombin ratio/international normalized ratio; RBC, red blood cell; RDW, red cell distribution width; r-GT, r-glutamyltransferase; T3, triiodothyronine; T4, thyroxine; TG, triglyceride; TIBC, total iron binding capacity; TSH, thyroid-

stimulating hormone; UA, uric acid; UTI, urinary tract infection; V5, oriented to verbal response of GCS and no significant respiratory distress and bulbar impairment ; VE, difficulty in evaluating GCS due to endotracheal intubation indicating severe bulbar impairment or respiratory distress; VLDL, very low density lipoprotein; VT, difficulty in evaluating GCS due to tracheostomy indicating severe bulbar impairment or respiratory distress; WBC, white blood cell. A *p*-value below 0.05 was considered statistically significant. $^{*}p < 0.05$; $^{**}p < 0.01$; [a]Fisher's exact test; [b]Two-Sample t test. Unless otherwise reported, qualitative values were expressed as percentage (n/total, %), quantitative values were median (interquartile range IQR).
