# Supplementary material for: Myasthenia gravis and independent risk factors for recurrent infection: a retrospective cohort study
Source: BMC Neurol. 2023 Jul 3;23:255. doi: 10.1186/s12883-023-03306-3 (PMC10316583; doi:10.1186/s12883-023-03306-3)
Supplement: Supplementary file 4 — Additional file 4. Univariate analysis via binary logistic regression of variables associated with recurrent infection of MG (full version). [file 12883_2023_3306_MOESM4_ESM.pdf]

Supplementary Table 4. Univariate analysis via binary logistic regression of variables associated with recurrent infection of MG (full version).

	Odds ratio	95% CI	*P-value*
**Male**	1.17	0.71-1.94	0.536
**Age**	1.02	1.00-1.03	0.028*
**Respiratory and bulbar function**			
V5	0.34	0.17-0.67	0.002**
V4	0.92	0.17-5.09	0.919
V3	NA	NA	NA
V2	0.00	0.00-.	1.000
V1	3057242713	0.00-.	0.999
VA	0.00	0.00-.	1.000
VE	3.57	1.56-8.16	0.003**
VT	1.84	0.11-29.78	0.667
**Motor scale (MRC)**			
Right deltoid	0.82	0.62-1.08	0.149
Left deltoid	0.80	0.59-1.08	0.139
Right iliopsoas	0.84	0.67-1.06	0.147
Left iliopsoas	0.82	0.66-1.04	0.097
Right deltoid and iliopsoas	0.90	0.79-1.03	0.130
Left deltoid and iliopsoas	0.89	0.78-1.02	0.099
Bilateral deltoid	0.89	0.76-1.03	0.120
Bilateral iliopsoas	0.91	0.80-1.02	0.106
Bilateral deltoid and iliopsoas	0.94	0.88-1.01	0.101
**Concomitant diseases**			
Hypertension	1.24	0.71-2.16	0.457
Diabetes mellitus	2.61	1.32-5.16	0.006**
Dyslipidemia	1.15	0.37-3.63	0.807
Congestive heart failure	0.51	0.11-2.52	0.412
Coronary artery disease	0.73	0.14-3.82	0.707
Atrial fibrillation	0.92	0.17-5.09	0.919
Deep vein thrombosis	9.62	1.11-83.54	0.040*
Asthma	2.81	0.46-17.09	0.263
Chronic obstructive pulmonary disease	0.92	0.08-10.23	0.943
Interstitial lung disease	2992879709	0.00-.	1.000
Gastroesophageal reflux disease	0.73	0.14-3.82	0.707
Peptic ulcer disease	1.91	0.69-5.26	0.211
Hepatitis B	0.45	0.05-4.11	0.481
Hepatitis C	0.00	0.00-.	0.999
Liver cirrhosis	1.86	0.37-9.40	0.453
Acute kidney injury	0.92	0.08-10.23	0.943
Chronic kidney disease	0.73	0.14-3.82	0.707

	Odds ratio	95% CI	*P-value*
End stage renal disease	0.00	0.00-.	1.000
Benign Prostatic Hyperplasia	0.92	0.17-5.09	0.919
Hyperthyroidism	0.48	0.13-1.78	0.274
Hypothyroidism	3.72	0.33-41.60	0.286
Cushing syndrome	0.61	0.06-5.92	0.667
Addison's disease	4.78	0.91-25.12	0.065
Gout	0.45	0.05-4.11	0.481
Myositis	0.92	0.08-10.23	0.943
Systemic lupus erythematosus	1.23	0.20-7.47	0.824
Iron deficiency anemia	3.78	0.68-21.04	0.129
Thymic cancer	2.22	1.17-4.19	0.014*
**Duration of hospitalization**	1.03	1.01-1.05	0.006**
**Foley catheterization**	0.61	0.335-1.04	0.069
**Ventilator dependency**	0.77	0.44-1.37	0.375
**Nasogastric tube**	0.86	0.50-1.49	0.592
**Central venous catheter**	0.61	0.33-1.13	0.117
**Plasmapheresis**	1.83	1.01-3.30	0.045*
**Seropositivity of AChR antibody**	1.89	0.98-3.68	0.059
**Laboratory data**			
WBC	1.04	0.98-1.10	0.168
Neutrocyte	1.02	1.00-1.03	0.097
Lymphocyte	0.98	0.96-1.00	0.116
Monocyte	1.01	0.92-1.12	0.786
Eosinophil	0.98	0.85-1.14	0.830
Basophil	1.02	0.49-2.12	0.955
RBC	0.76	0.54-1.09	0.133
Hct	0.96	0.92-1.01	0.085
Hb	0.88	0.78-1.00	0.037*
MCH	0.97	0.90-1.05	0.467
MCHC	0.84	0.69-1.01	0.066
MCV	1.00	0.97-1.03	0.893
RDW	1.05	0.93-1.20	0.420
Platelet	1.00	1.00-1.00	0.541
BUN	1.00	0.99-1.02	0.731
Creatinine	1.08	0.90-1.30	0.385
Na	0.97	0.90-1.04	0.414
K	0.49	0.28-0.85	0.011*
Ca	0.73	0.48-1.11	0.139
P	0.91	0.66-1.26	0.566
Mg	0.16	0.03-0.89	0.037*
CL	1.10	0.98-1.23	0.119

	Odds ratio	95% CI	*P-value*
AST	0.99	0.98-1.01	0.293
Bilirubin total	0.95	0.38-2.39	0.908
Bilirubin direct	10.05	0.49-204.63	0.133
ALP	1.01	1.00-1.01	0.241
r-GT	1.01	1.00-1.01	0.216
Albumin	0.63	0.41-0.96	0.030*
PT/INR	1.85	0.36-9.48	0.461
APTT	1.08	1.01-1.16	0.026*
D-dimer	1.00	1.00-1.00	0.910
TG	1.00	1.00-1.00	0.660
Cholesterol	1.00	0.99-1.01	0.871
LDL	1.01	1.00-1.02	0.282
VLDL	0.99	0.94-1.04	0.674
HDL	1.02	0.98-1.06	0.341
Lupus anticoagulant	1.00	0.94-1.06	0.961
CK	1.00	1.00-1.00	0.468
LDH	1.00	0.99-1.00	0.194
CRP	1.00	0.99-1.00	0.577
ANA	1.08	0.32-3.65	0.901
C3	0.98	0.96-1.01	0.217
C4	0.99	0.92-1.06	0.762
Fe	0.99	0.95-1.03	0.605
Ferritin	1.00	0.99-1.00	0.336
TIBC	1.00	0.99-1.01	0.619
HbA1c	1.24	0.93-1.65	0.137
Glucose	1.00	1.00-1.01	0.108
TSH	1.04	0.97-1.11	0.309
Free T4	1.20	0.42-3.43	0.740
T3	1.00	0.98-1.02	0.904
T4	0.93	0.67-1.28	0.651
Cortisol	0.96	0.91-1.00	0.065
UA	0.96	0.78-1.18	0.682

AChR, acetylcholine receptor; ALP, alkaline phosphatase; ANA, antinuclear antibody; APTT, activated partial thromboplastin time; AST, aspartate aminotransferase; BUN, blood urea nitrogen; Ca, calcium; CK, creatine kinase; CL, chloride; CRP, C-reactive protein; Fe, iron; Free T4, free thyroxine; GCS, Glasgow coma scale; Hb, hemoglobin; HbA1c, glycohemoglobin; Hct, hematocrit; HDL, high density lipoprotein; K, potassium; LDH, lactate dehydrogenase; LDL, low density lipoprotein; MCH, mean corpuscular hemoglobin; MCHC, mean corpuscular hemoglobin concentration; MCV, mean corpuscular volume; MG, myasthenia gravis; Mg, magnesium; MRC, Medical Research Council scale; NA, not applicable; Na, sodium; P, phosphorus; PT/INR, prothrombin ratio/international normalized ratio; RBC, red blood cell;

RDW, red cell distribution width; r-GT, r-glutamyltransferase; T3, triiodothyronine; T4, thyroxine; TG, triglyceride; TIBC, total iron binding capacity; TSH, thyroid-stimulating hormone; UA, uric acid; V5, oriented to verbal response of GCS and no significant respiratory distress and bulbar impairment ; VE, difficulty in evaluating GCS due to endotracheal intubation indicating severe bulbar impairment or respiratory distress; VLDL, very low density lipoprotein; VT, difficulty in evaluating GCS due to tracheostomy indicating severe bulbar impairment or respiratory distress; WBC, white blood cell. Risk for recurrent infection in MG patients was presented as odds ratio. 95% CI, 95% confidence interval. A *p*-value below 0.05 was considered statistically significant. $^{*}p < 0.05$; $^{**}p < 0.01$.
